# Dietary shifts and social interactions drive temporal fluctuations of the gut microbiome from wild redfronted lemurs

**DOI:** 10.1038/s43705-021-00086-0

**Published:** 2022-01-19

**Authors:** Tatiana Murillo, Dominik Schneider, Claudia Fichtel, Rolf Daniel

**Affiliations:** 1 Behavioral Ecology and Sociobiology Unit, German Primate Center, Göttingen, Germany; 2 Genomic and Applied Microbiology and Göttingen Genomics Laboratory, Institute of Microbiology and Genetics, Georg-August-University of Göttingen, Göttingen, Germany

**Keywords:** Microbiome, Microbial ecology, Microbial ecology

## Abstract

Animals living in highly seasonal environments adapt their diets accordingly to changes in food availability. The gut microbiome as an active participant in the metabolization of the host’s diet should adapt and change with temporal diet fluctuations, but dietary shifts can be short-term and, hence, difficult to detect in cross-sectional studies. Therefore, we performed a longitudinal study combining repeated sampling of fecal samples with observations of feeding behavior in wild redfronted lemurs. We amplified taxonomical marker genes for assessing the bacteria, archaea, protozoa, helminths, and fungi, as well as the active bacterial community inhabiting their gut. We found that the most abundant protozoans were *Trichostomatia* and *Trichomonadida*, and the most abundant helminths were *Chromadorea*. We detected known members of the gut mycobiome from humans but in low abundances. The archaeal community is composed only of members of *Methanomethylophilaceae*. The predominant phyla in the entire bacterial community were *Bacteroidota* and *Firmicutes* while the most abundant genera harbor so far unknown bacteria. Temporal fluctuations at the entire community level were driven by consumption of fruits and flowers, and affiliative interactions. Changes in alpha diversity correlated only with the consumption of flowers and leaves. The composition of the entire and active bacterial community was not significantly different, but the most abundant taxa differed. Our study revealed that monthly changes in the bacterial community composition were linked to fruit and flower consumption and affiliative interactions. Thus, portraying the importance of longitudinal studies for understanding the adaptations and alterations of the gut microbiome to temporal fluctuations.

## Introduction

The gut microbiome is a complex fluctuating microbial ecosystem comprising prokaryotic and eukaryotic microorganisms playing a pivotal role in immunity, physiology, metabolism, and susceptibility to disease of the host [1, 2]. Investigations of factors driving these fluctuations help to understand how this ecosystem adapts to the changing conditions, and the potential effects these variations have on the health and fitness of their hosts [2–4].

Essential nutrient cycling processes of the gut ecosystem occur between the host diet, the microorganisms, and the host itself [1, 2, 4]. Bacteria catalyze the fermentation of dietary fiber and starch into short-chain fatty acids and monosaccharides taken up by the host and other microorganisms [1, 2]. They also provide ammonia for protein synthesis by metabolizing essential and non-essential amino acids [2]. The host diet shapes the microbial gut communities and the presence of certain microorganisms is crucial for proper degradation and uptake of nutrients from diet and the resilience of the gut ecosystem [1, 2, 4]. Therefore, the gut microbial ecosystem of wild animals living in highly seasonal environments should be capable of adapting to dietary changes following fluctuations in food availability and seasonality [3–5]. Research in wild mice (*Apodemus sylvaticus*), Tibetan macaques (*Macaca thibetana*) and pandas (*Ailuropoda melanoleuca*) found marked seasonal variations in the gut microbiome composition and diversity associated with environmental fluctuations affecting food availability [5–7]. Furthermore, cross-sectional studies in black howler monkeys (*Alouatta pigra*), white faced capuchins (*Cebus capucinus*), and Verreaux’s sifakas (*Propithecus verreauxi*) determined these fluctuations correlate with changes in foraging and feeding behaviors [8–10]. Nonetheless, by sampling only representative months of each season, short-term dietary and gut microbiome shifts might be undetected [4]. In the Hadza hunter-gatherers, a seasonal cycling of the gut microbiome following seasonal changes in their diets between fruit foraging and hunting was detected [11]. Longitudinal studies in great apes (*Gorilla gorilla gorilla*, *Gorilla beringei beringei* and *Pan troglodytes troglodytes*) and geladas (*Theropithecus geladas*) determined seasonal fluctuations of the gut microbiome correlate with rainfall, temperature, and food availability [12, 13]. While a time series study in baboons (*Papio cynocephalus)* detected a highly dynamic gut microbiome varying according to the group’s diet, rainfall, and the quality of the water sources [14]. Thus, enhancing the importance of time series analysis in wild animals to determine how the gut microbial communities adapt to seasonal changes [4, 8, 12, 14].

To our knowledge, all taxonomic profiling studies in wild animals focus on the amplification of 16 S rRNA gene from DNA, hence studying the entire community. This approach can be biased by the number of 16 S rRNA operons and the presence of dormant or dead cells in the sample [15]. Conversely, when amplifying the 16 S rRNA transcripts, only the bacterial community that is actively replicating is investigated, providing insights into the potentially active community [16]. This approach can provide better proxies into the functional metabolic changes that the gut microbiome undergoes as a response to seasonality [2, 4].

We performed a longitudinal analysis of the entire and active gut bacterial community in a wild primate, the redfronted lemur (*Eulemur rufifrons*). Their habitat, Kirindy Forest in Madagascar, is highly seasonal having a cold dry season from April to October and a warm rainy season from November to March [17, 18]. These environmental conditions cause changes in the availability of food and water sources, posing adaptive challenges for these animals [19–21]. Redfronted lemurs are mainly frugivorous but consume leaves, and flowers following seasonal fluctuations, and adjust their drinking behavior according to the available water sources [20, 21]. Hence, these redfronted lemurs are a suitable study system to characterize temporal fluctuations in the gut microbiome composition. Moreover, they possess a high eukaryotic parasite richness with variations in their monthly prevalence as detected from morphological studies, suggesting complex prokaryotic and eukaryotic interactions occur in their guts [18, 22, 23]. However, their gut mycobiome is still unexplored despite its potential metabolic importance [24, 25].

For 1 year, we collected up to three fecal samples per month for each individual and conducted regular animal focal observations to determine their dietary composition and affiliative interactions. Since previous research suggested that social group and home range can also impact the gut microbiome [10, 26], we studied only one group consisting of five individuals to control for these potential confounding factors. To characterize the microbiome composition, we assessed the entire and active bacterial community as well as other inhabitants of the gut, including *Protozoa*, helminths, *Fungi*, and *Archaea*. We hypothesize that by using a longitudinal approach, we [1] determine temporal fluctuations in composition and diversity of the bacterial entire and active community correlate to monthly changes in diet and affiliative interactions, [2] find no significant differences between the entire and the active bacterial communities, and [3] detect temporal changes in the abundances of the eukaryotic community.

## Methods

### Sample, behavioral, and environmental data collection

This study was conducted at the research station of the German Primate Center in Kirindy Forest, Western Madagascar (44°39′E, 20°03′S) from May 2018 to April 2019 [17]. Samples and data were collected over 1 year from five redfronted lemurs belonging to the same group; three adult females (FLucF, FTorF, and FMayF), one juvenile female (FBonF) and one adult male (FCaiM) (Supplementary Table S1). Fecal samples were collected in RNAlater (Thermofisher Scientific, Massachusetts, USA) from the forest floor immediately after defecation between 7:30 and 11:00, stored at −20 °C in the field station and later at −80 °C in Germany. A total of 142 samples were collected, with an average of two samples per individual per month (Supplementary Table S1). Behavioral data was collected by continuous focal observations for 30 min in the morning (7:30–11:00) and afternoon (14:00–17:00). Feeding behaviors were recorded by protocolling their duration and the ingested food item (leaves, flowers, or fruits). For affiliative interactions, we protocolled the duration of grooming and body contact behavior. Environmental data (daily temperature and precipitation) were collected at the field station with a Tropos data logger (Lambrecht meteo, Göttingen, Germany).

### DNA extraction and amplification of taxonomic marker genes

DNA extractions were performed with the PowerSoil DNA isolation kit (Qiagen, Hilden, Germany) using 150 mg fecal sample following the manufacturer´s instructions but including a bead beating step of 6.5 m/s and 24 × 2 for 20 s using FastPrep-24^TM^5G (MP Biomedicals, California, USA). PCR reactions for all taxonomical marker genes were performed in triplicates with the primers and thermocycling protocols listed in the Supplementary Table S2 and included a negative control without DNA template and a positive control [27–32]. Triplicates per sample were pooled equimolar, purified, and sequenced as in [33].

### RNA extraction and cDNA synthesis

RNA was extracted from 250 mg fecal sample using the RNeasy Power Microbiome kit (Qiagen) following the manufacturer´s instructions, and according to the protocol from [33].

### Bioinformatic processing of amplicon data

Paired-end reads were quality-filtered with Fastp0.20.0 [34] using default settings with the addition of an increased per base phred score of 20, base pair corrections by overlap (-c), as well as 5′- and 3′-end read-trimming with a sliding window of 4, a mean quality of 20 and minimum sequence length of 50 bp. Quality-controlled reads were merged with PEARv0.9.11 [35] and primer-clipping was performed with cutadapt2.5 [36] with default settings. VSEARCH2.14.1 [37] was used for size-sorting, size-filtering (16 S rRNA ≥ 300 bp; 18 S rRNA ≥ 250 bp; ITS2 ≥ 140 bp) and dereplication. The sequences were denoised with UNOISE3 [38] using default settings and chimeras were removed with UCHIME3 (*de novo* followed by reference-based) [39] leading to the final set of amplicon sequence variants (ASVs). Then all reads were mapped against the ASVs and taxonomy was assigned with a minimum identity of 90% using BLAST2.9.0 + [40] against different databases according to the taxonomical marker gene. The databases were SILVA SSU 138 NR [41] for 16 S rRNA, PR^2^ SSU rRNA [42] for 18 S rRNA and UNITE 8.2 [43] for ITS2. Best hits were only accepted if $\big( {\frac{{\% identity + \% coverage}}{2}} \big) \ge 93$ following the recommendation of SILVA database [41]. Best blastn hit identity for bacterial species <98.7% or genus <94.5% were corrected to unclassified [44]. Functional predictions were performed using Faprotax1.2.3 [45] for the bacterial 16 S rRNA gene data after beforementioned filters were applied. All sequencing statistics are presented in Supplementary Table S3.

### Data visualization and statistical analysis

Data visualization and statistical analysis were performed using Rv3.6.2 [46] and RStudiov1.20.5033 [47] by using the packages ampvis2 [48], ape [49], stringr [50], reshape2 [51], viridis, data.table [52], tidyverse [53], and ggplot2 [54]. Datasets for barcharts, heatmaps, and linecharts were normalized using GMPR [55], whereas data was rarefied for diversity and multivariate analysis (Supplementary Table S3). A phylogenetic tree was generated by aligning all sequences with MAFFTv7.407-1 [56] at 100 iterations, calculated using FastTreeMPv2.1.7 [57] and midpoint-rooted using FigTree v1.4.4 [58] for estimating Faith´s phylogenetic diversity (PD) with the package picante [59].

For the 18 S rRNA gene amplicon analysis of eukaryotic parasites and symbionts, samples with <9000 reads were excluded leaving 115 samples. ASVs from the kingdoms previously reported as inhabitants of the gastrointestinal tract of animals: *Cercozoa*, *Ciliophora*, *Metazoa*, *Apicomplexa*, *Lobosa*, *Conosa,* and *Metamonada* were analyzed [23, 60]. For the ITS2 dataset samples with <7000 reads after quality-filtering samples were removed leaving 125 samples for analysis.

#### ANCOM analysis to estimate differential taxa between seasons

To determine bacterial genera with significant different relative abundances between seasons, we used ANCOM 2.1 [61] and the packages exactRankTests [62], nlme [63], compositions [64], and readr [65] by using the repeated measures model with season as main variable and individual as random effect, and 0.7 as threshold of the *W* statistic.

#### Multivariate analysis to study temporal changes in β-diversity

Principal coordinate analyses (PCoA) using weighted UniFrac distances (WUnifrac) [66, 67] were calculated in ampvis2 [48]. To test for correlations of the behavioral and environmental variables an environmental fit with 999 permutations was calculated and corrected for repeated sampling by using strata as individual with vegan [66]. A PERMANOVA test was calculated with the adonis function from the vegan package to test for significant differences between individual β-diversity calculated as WUnifrac. Mantel tests using Spearman correlations were calculated with the vegan package to estimate correlations between β-diversity from WUniFrac distances and time between sample collection.

#### Linear mixed model for estimating the effects on bacterial composition

The effects of feeding behaviors and affiliative interactions on the bacterial composition of the entire bacterial community were tested by fitting a Linear Mixed Model (LMM) with lme4 [68]. The model included monthly feeding rates (min/h) on fruits, leaves, or flowers, and affiliative interactions (min/h) per individual as test predictors, and mean monthly precipitation (mm) as control predictor. Taxa with abundances <0.5% in a sample were removed to account for index hopping during sequencing [69]. To deal with data compositionality, the microbial proportions of each sample were centered log-ratio transformed [70]. The random intercepts effects of individual, taxon, sample, and taxon nested within individual (taxon-individual) were included, the latter to account for individual specific microbial compositions. Random slopes for all predictors in taxon, individual, and taxon-individual were included, excluding flower feeding rates for taxon-individual [71]. Parameters for the correlations between random intercepts and slopes within taxon and taxon-individual were included [71] but not within individual because they were unidentifiable [72]. Assumptions of normally distributed and homogeneous residuals were checked visually with QQ-plots of residuals and residuals plotted against fitted values which revealed no obvious deviations. No issues of collinearity were detected by calculating Variance Inflation Factors using car [73] on a model lacking the random effects (maximum: 1.203). The crucial terms in this model were the random slopes within taxon representing the taxon-specific effects of the test predictors and were tested with a permutation test by shuffling the labels of taxa within sample [74, 75]. As a test statistic, we used the difference between the log likelihoods of the full model and simpler models. One of the simpler models lacked all random slopes within the sample except that of precipitation allowing a full-null model comparison by testing the combined effects of all test predictors. The others lacked the individual random slopes (except precipitation) within taxon allowing to test their individual contribution. A total of 1000 permutations including the original data as one permutation were conducted, and *p*-values were calculated as the proportion of permutations that revealed a test statistic at least as large as that of the original data. If an individual random slope effect was significant, then the effect of the respective predictor differs between taxa. The 20 taxa differing most from the average effect across all taxa, meaning they had the largest absolute values of the respective Best Linear Unbiased Predictors (BLUPs), were inspected [76]. Model stability was assessed by dropping individuals one at time, fitting the full model to each of the subsets, and then comparing the estimates derived with those obtained for the full model revealing it was acceptable. Residuals for each combination of taxon and predictor were plotted verifying the presence of linear trends.

#### Linear mixed models for estimating effects on alpha diversity

The effects of feeding behaviors and affiliative interactions on alpha diversity for the entire and active bacterial community were estimated by fitting a LMM using lme4 [68], MuMIn [77], and visualized with sjPlot [78]. The response variable was PD, which was log-transformed for the model of the active community. Affiliative interactions were log-transformed to achieve a more symmetrical distribution and avoid influential cases, and all predictors were z-transformed to facilitate model convergence. We included individual identity as a random intercept effect and the random slopes of all fixed effects into individual identity to keep the type I error at the nominal level of 5% [71]. For estimating the significance of the test predictors, a null model excluding the test predictors was calculated and then compared to the full model using a likelihood ratio test. We determined the effect of single fixed effects using likelihood ratio tests comparing the full model with reduced models removing one fixed effect at a time [71]. Model assumptions and collinearity (DNA: 1.203; RNA: 1.205) were checked as in the LMM for bacterial composition with no obvious deviations from these assumptions. Model stability was assessed as described above.

#### Procrustes analysis

Procrustes analysis and significance testing with protest were performed using vegan [66] to test for correlations between the plant material detected from the 18 S rRNA gene amplicons and the entire bacterial community from calculated PCoAs of Bray Curtis dissimilarity matrices in ampvis2 [48]. Only those samples with >1000 reads for *Archaeplastida* were analyzed, leaving 97 samples after rarefaction. The same test was used to determine significant differences between the composition of the entire and active bacterial community from the PCoAs from WUnifrac distances. A summary of all statistical results is depicted in Supplementary Table S4.

### Gene alignments and phylogenetic tree from eukaryotic data

Sequence alignments were done with MUSCLE [79] with UPGMA and default settings. Phylogenetic trees were calculated with the Maximum Likelihood method, Tamura-Nei model, and 1000 bootstrap in MEGA X [80]. The 18S rRNA gene and ITS2 sequences from representative nematodes and *Fungi* were retrieved from GenBank database [81].

### Data deposition

The 16 S rRNA gene and transcripts, 18 S rRNA gene, and ITS2, paired-end raw reads were deposited in the National Center for Biotechnology Information Sequence Read Archive (SRA) under the Bioproject PRJNA694983. SRA numbers are in Supplementary Table S1.

## Results

### Composition of the redfronted lemur gut microbiome

**Fig. 1:**
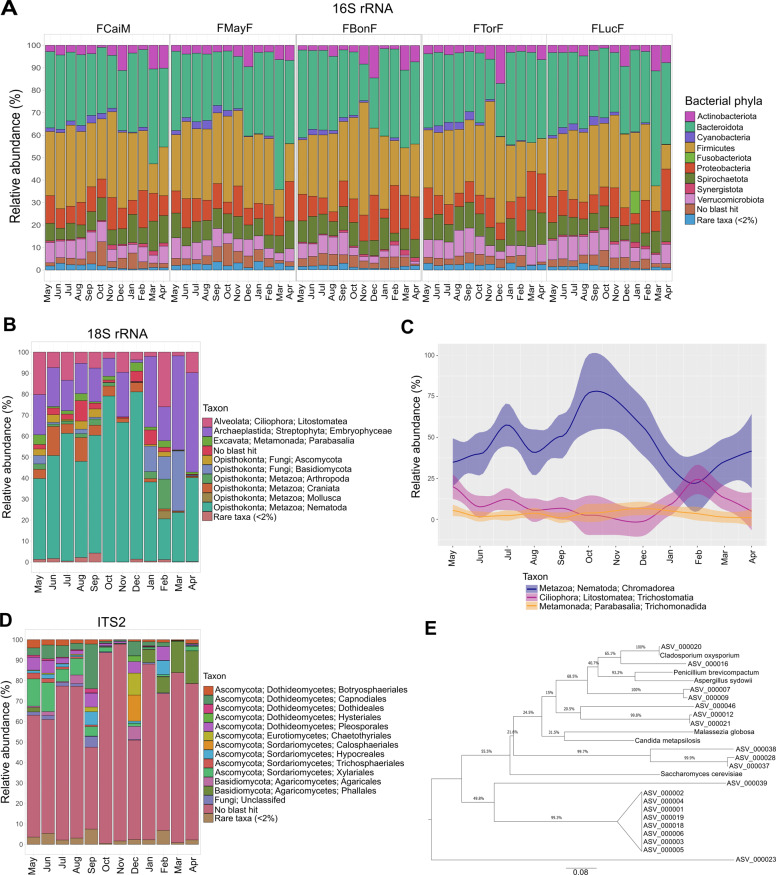
Prokaryotic and eukaryotic communities from the gut of redfronted lemurs during the study period from May 2018 until April 2019. **A** Monthly relative abundances of bacterial phyla for the five studied individuals as determined from 16 S rRNA gene sequencing. Bar charts depict relative abundances of bacterial phyla from normalized counts for each individual per month. All phyla with abundances <2% were grouped as rare taxa. **B** Eukaryotic organisms detected in the fecal samples through 18 S rRNA gene sequencing. Bar charts show monthly relative abundances of eukaryotic classes from normalized counts. All phyla with abundances <2% were grouped as rare taxa. **C** Monthly fluctuations in the relative abundances of *Chromadorea*, *Trichostomatia*, and *Trichomonadida*. Linecharts depict relative abundances of normalized counts of the detected eukaryotic parasites or endosymbionts in the fecal samples. **D** Fungal organisms detected in the fecal samples through ITS2 sequencing. Barcharts display monthly relative abundances of fungal orders from normalized counts. All taxa with abundances <2% were unified as rare taxa. **E** Maximum likelihood phylogenetic tree of the unclassified ITS2 ASVs against representative *Fungi*.

The most abundant bacterial phyla in the five redfronted lemurs were constant throughout the sampling period with varying relative abundances; these were *Bacteroidota* (30.6% ± 7.6), *Firmicutes* (30.0% ± 8.2), *Proteobacteria* (12.3% ± 6.5), *Spirochaetota* (8.7% ± 2.5) and *Verrucomicrobiota* (6.3% ± 2.2) (Fig. 1A and Supplementary Table S5). These were consistent for all individuals exempting an increase of *Firmicutes* (55.7%) and *Proteobacteria* in February for FBonF, and an increase of *Fusobacteriota* (9.9%) in January for FLucF.

The taxa detected from the amplification of the 18 S rRNA gene were *Metazoa* (56.9% ± 22.7), *Streptophyta* (21.3% ± 13.2), *Fungi* (6.6% ± 6.1), *Ciliophora* (9.6% ± 7.2), and *Metamonada* (1.9% ± 1.5) with a total of 3.1% ± 2.9 unclassified reads (Fig. 1B and Supplementary Table S6). The most abundant orders previously reported as eukaryotic parasites detected were *Chromadorea*, *Trichostomatia,* and *Trichomonadida* (Fig. 1C). *Chromadorea* highest abundances were in October (94.4%) and lowest in February (40.0%). While *Trichostomatia* increased in February (53.6%), and *Trichomonadida* in May (7.3%). Overall, ASVs classified as *Chromadorea* showed high diversity, indicating a diverse nematode community (Supplementary Fig. S1).

To study fungal gut communities, we analyzed the ITS2 region. A total of 71% ± 16.8 of sequences were unclassified to Kingdom, thus demonstrating a lack of information from Malagasy fungal organisms in databases (Fig. 1D and Supplementary Table S7). When studying the gut mycobiome the separation between symbionts and environmental fungi using metagenomic approaches is challenging [24, 82]. Especially in redfronted lemurs, who feed on *Fungi* and plants, which potentially harbor fungal pathogens. Thus, we extracted only those fungal genera described before as gut symbionts [24]. We detected these genera in relative abundances <1%: *Cryptococcus*, *Agaricus*, *Candida*, *Saccharomyces*, *Malassezia,* and *Clavispora* whereas other genera like *Cladosporium*, *Aspergillus*, *Fusarium,* and *Penicillium* were present in relative abundances >1%. In a phylogenetic analysis calculated from the 20 most abundant unclassified ASVs against ITS2 sequences from some of the fungal genera described as inhabitants of the gut mycobiome only one ASV was phylogenetically related to *Cladosporium* (Fig. 1E). Also, no similar sequences were detected in the NCBI database.

The archaeal community was assessed with 16 S rRNA gene analysis in a smaller set of samples using two different sets of primers aiming to recover sequences of different lineages. In both cases, only *Methanomethylophilaceae* was identified (Supplementary Tables 8 and 9). Thus, the archaeal community has a low diversity and comprises members also known from the gut of great apes and humans [83, 84].

### Temporal variations of the entire gut bacterial community composition

**Fig. 2:**
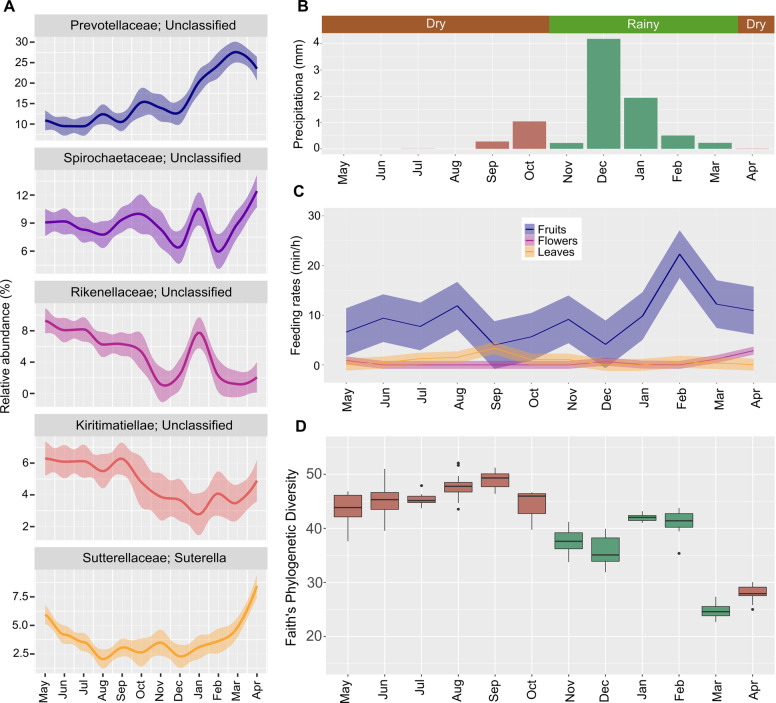
Monthly fluctuations in most abundant bacterial genera and alpha diversity detected in fecal samples from redfronted lemurs from May 2018 to April 2019. **A** Top 5 most abundant bacterial genera and their monthly changes for all studied individuals. Line charts display relative abundances from normalized counts. **B** Mean monthly precipitation calculated from records of daily precipitation and seasons from the study period. **C** Monthly feeding rates on fruits, leaves, and flowers determined through behavioral focal observations. **D** Monthly variations in alpha diversity measured by Faith’s Phylogenetic Diversity Index of all studied individuals.

The five most abundant genera comprise mostly novel organisms for which only classification at the family level was possible (Fig. 2A). These genera belong to the four families of *Prevotellaceae* (14.6% ± 7.4), *Spirochaetaceae* (8.9% ± 3.1), *Rikenellaceae* (5.7% ± 4.1) and *Kiritimatiellae* (5.1% ± 2.4). The fifth most abundant genus was *Sutterella* (3.9% ± 2.3). All showed monthly fluctuations in their abundances, which were not always consistent among individuals (Supplementary Fig. S2). The top 20 most abundant genera also presented monthly and individual differences in their relative abundances (Supplementary Fig. S3). A PERMANOVA test confirmed the β-dissimilarities were significantly different between individuals (*p* < 0.002).

Seasons were defined following previous publications [17], however, during our study rainfall increased at the end of the dry season (Fig. 2B), and feeding behaviors varied across months (Fig. 2C). Alpha diversity increased during the dry season with a maximum between August and October (Fig. 2D). The PD value fluctuated during the whole rainy season and was lower compared to the dry season. Monthly alpha diversity changes followed the same pattern in all individuals (Supplementary Fig. S4).

**Fig. 3:**
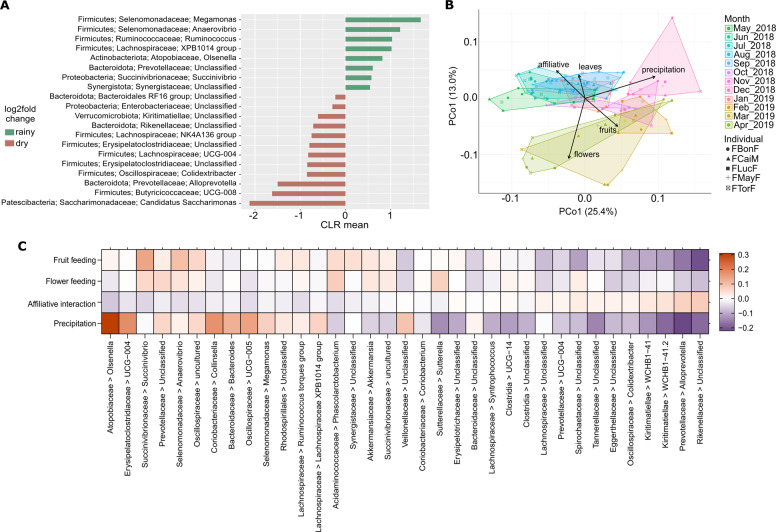
Seasonal variations of bacterial genera, beta diversity, and composition of the entire gut bacterial community of redfronted lemurs from May 2018 until April 2019. **A** Log2f fold changes in the mean abundances of bacterial genera between dry and rainy season as detected with ANCOM 2.1. **B** PCoA based on weighted Unifrac of the bacterial community and environmental fit analysis depicting significant correlations between temporal fluctuations in beta diversity and the environmental, diet and social factors investigated. **C** Heatmap showing the 20 bacterial genera for which taxon-specific effects differed most from the average across all taxa as detected in a LMM estimating the effects of diet and affiliative interactions on community composition. The image displays the test predictors for which an effect was detected, feeding on flowers and fruits, and affiliation rates. Precipitation was included as the control predictor. Positive effects are depicted with orange, whereas negative effects are colored in purple.

ANCOM analysis revealed that 75 genera showed significant differential abundance between dry and rainy seasons (Supplementary Fig. S5). We focused on taxa classified with relative abundances ≥1% (Fig. 3A).

Mean monthly precipitation, consumption of fruits, leaves, and/or flowers and the rate of affiliative interactions correlated to the temporal variations in β-diversity. Samples from the dry season clustered together unlike the samples from the rainy season (Fig. 3B), and season (*p* = 0.001), precipitation (*p* = 0.001), feeding on fruits (*p* = 0.003), leaves (*p* = 0.044), flowers (*p* = 0.001), and affiliative interactions (*p* = 0.006).

The LMM detected taxon-specific effects (full-null model comparison; permutation test: *p* = 0.001) of flower (*p* = 0.001) and fruit feeding (*p* = 0.001), and affiliative interactions (*p* = 0.043) on the overall bacterial community composition (Supplementary Table S10) the following: exhibited significant correlations. We thus inspected the 20 taxa for which the taxon-specific effect deviated most from the average effect across all taxa for each significant predictor (Fig. 3C and Supplementary Fig. S6–9).

A time series analysis of WUnifrac distances against time between sample collection confirmed temporal variations on individual level (Supplementary Fig. S10 and Supplementary Table S11). Thus, the longer the timespan between the samples, the more dissimilar were the gut bacterial communities.

**Fig. 4:**
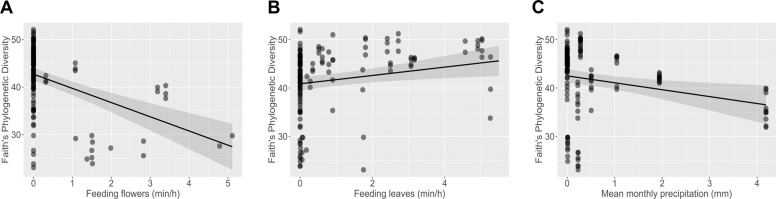
Environmental and dietary factors driving the monthly fluctuations in alpha diversity of the entire bacterial community measured with the Faith’s Phylogenetic Diversity index. **A** Monthly rates of flower consumption (min/h) correlate negatively with alpha diversity. **B** Monthly rates of leaves feeding (min/h) correlate positively with a higher alpha diversity. **C** Mean monthly precipitation correlates negatively with alpha diversity. The effects of diet, affiliation rates and precipitation were determined with a LMM.

The LMM for the alpha diversity (full-null model comparison: *p* = 0.003) detected an effect of feeding on leaves (*p* = 0.055, Fig. 4B) which correlated with an increase in PD, while the rates of flower consumption and mean monthly precipitation correlated negatively with PD (flowers: *p* = 0.002; Fig. 4A; monthly rainfall: *p* = 0.039, Fig. 4C, Supplementary Table S12). An effect of dietary changes on bacterial community composition was further confirmed by significant correlations from the plant diet deduced from the 18 S rRNA gene analysis (Supplementary Fig. S11A) to the fluctuations of the bacterial community (Supplementary Fig. S11B) (*p* = 0.001; Supplementary Fig. S11C).

### Potential active bacterial community in the redfronted lemur gut

**Fig. 5:**
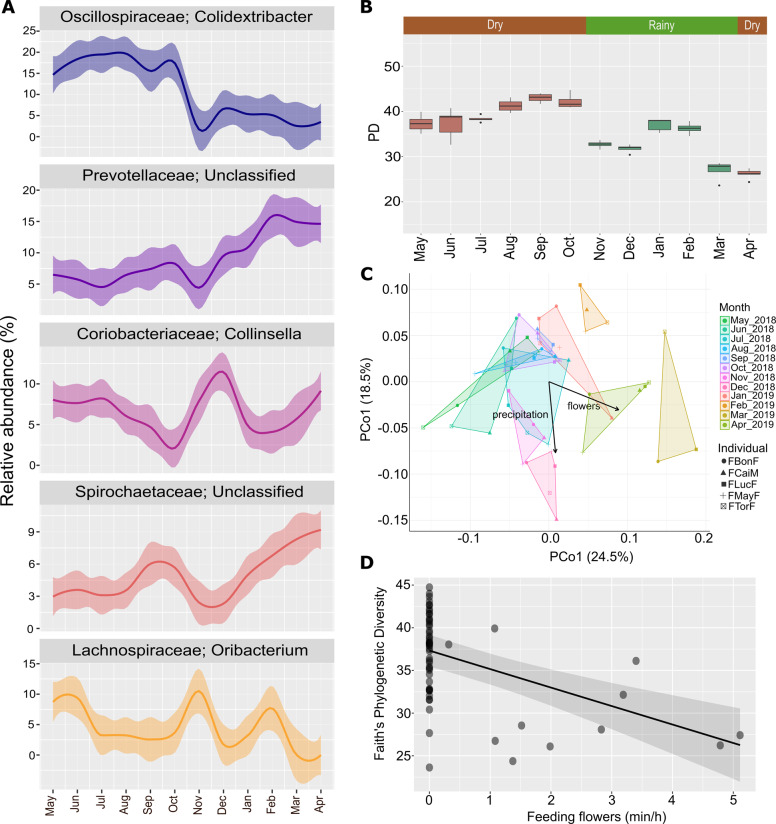
Monthly fluctuations in the bacterial composition and alpha diversity of the active bacterial community in fecal samples from redfronted lemurs from May 2018 to April 2019. **A** Top five most abundant bacterial genera and their monthly changes for all studied individuals. Line charts display relative abundances from normalized counts. **B** Monthly variations in alpha diversity measured by Faith’s Phylogenetic Diversity Index in all studied individuals. **C** PCoA from WUnifrac of the bacterial community and environmental fit analysis depicting significant correlations between temporal fluctuations in beta diversity and the environmental, diet and social factors investigated. **D** Monthly rates of flower consumption (min/h) correlate negatively with alpha diversity. The effects of diet, affiliation rates and precipitation on alpha diversity were determined with an LMM.

The potentially active bacterial communities were analyzed from one sample per individual per month. The five most abundant phyla detected in the active community were *Firmicutes* (56.1% ± 13.1), *Bacteroidota* (16.5% ± 6.1), *Actinobacteriota* (9.9% ± 4.4), *Proteobacteria* (5.2% ± 2.2), and *Spirochaetota* (4.9% ± 2.2) (Supplementary Fig. S12A). The five most abundant genera were *Colidextribacter* (11.5% ± 8.1), *Prevotellaceae*—Unclassified (8.8% ± 4.8), *Collinsella* (6.7% ± 3.4), *Spirochaetaceae*—Unclassified (4.8% ± 2.8), and *Oribacterium* (4.5% ± 4.6) (Fig. 5A and Supplementary Fig. S12A). The top 20 most abundant genera were also investigated, which presented monthly and individual differences in their relative abundances (Supplementary Fig. S13).

The ANCOM analysis revealed that 40 genera exhibited significantly different relative abundances between seasons. Most exhibited abundances <1% or were only classifiable to order level (Supplementary Fig. S14) leaving only *Bacteroidales* group RF16 with lower abundances in the rainy season, whereas *Lachnospiraceae* group XPB1014 and *Fusobacterium* had higher abundances in the rainy season. PD was higher during the dry season and more variable during the months of the rainy season, like at the entire community level (Fig. 5B). The PCoA did not show seasonal clustering (Fig. 5C). However, the environmental fit analysis detected correlations of season (*p* = 0.007), feeding on flowers (*p* = 0.003) and precipitation (*p* = 0.013) with the monthly alterations of the bacterial community (Fig. 5C). Mantel correlation tests of the β-dissimilarities and the timespan between sample collection for each individual were significant (Supplementary Table 13). Only feeding on flowers (*p* = 0.002) was associated with an effect in alpha diversity correlating with a decrease (Fig. 5D, Supplementary Table S14).

For comparison of entire with active communities a PCoA from WUnifrac with the reduced sample size was calculated also at entire community level. Precipitation (*p* = 0.003) and feeding on fruits (*p* = 0.056) and flowers (*p* = 0.002) were significantly correlated (Supplementary Fig. S15A). The comparison between the PCoAs from the entire and active community with the protest test from the Procrustes analysis detected significant correlations (*p* = 0.001). Thus, they were not significantly different (Supplementary Fig. S15B).

Functional predictions performed for the active community assigned 51.7% of the ASVs to an entry of the Faprotax database. Chemoheterotrophy (21.6% ± 3.6) and fermentation (21.3 ± 3.7) were the most abundant metabolisms, with a peak during the rainy season from October to January coinciding with an increase in fruit feeding (Fig. 2C and Supplementary Fig. S16).

## Discussion

Our longitudinal approach coupled with a dense sampling regime and behavioral data allowed us to detect in detail the temporal fluctuations of the gut microbial communities from redfronted lemurs. We determined the entire bacterial community changed accordingly to a higher consumption of fruits and flowers, and variations in affiliative interactions. Hence, the bacterial community quickly adapted to monthly changes in the diet but also to the host social behavior. Moreover, we characterized the potentially active bacterial community, which also underwent temporal fluctuations that correlated but only to flower consumption. The overall composition of the entire and the active bacterial communities were not significantly different, but the most abundant genera differed. The eukaryotic communities also presented temporal fluctuations and includes undescribed organisms.

### Unknown genera inhabit the gut microbiome of redfronted lemurs

The most abundant bacterial phyla identified were *Bacteroidota* and *Firmicutes* similarly to other primates and humans [12, 85, 86]. *Spirochaetota* was also detected in high abundances, coinciding with previous reports from other primates and a cross-sectional study from the same species [86, 87]. A previous study in the same lemur species detected only low abundances of treponemes but higher abundances of *Cyanobacteria* and *Firmicutes* [87]. However, in this study, samples were not placed in preservation solution for a time span of 12 h, which might have altered the bacterial community [87].

The impossibility to classify the most abundant bacteria to taxonomic resolutions below family level highlights the presence of yet unclassified microorganisms in the gut of redfronted lemurs, as described in other non-human primates [86]. While the classifiable taxa are reported inhabitants of the gut from humans and other non-human primates [8, 11, 13, 86, 88]. Genera from *Prevotellaceae* and *Spirochaetaceae*, have been associated to plant-based diets providing pathways for their metabolization [11, 85, 86]. *Rikenellaceae* ferments carbohydrates and proteins [89]. Taxa from *Verrucomicrobiota* have been reported as mucin-utilizers [11]. Less is known about the metabolic role of *Sutterella*, a common inhabitant of the human gut [90].

### The potential active bacterial community has a lower alpha diversity and differs in the most abundant taxa

The potential active community had higher relative abundances of *Firmicutes* and a lower alpha diversity compared to the entire community. There are several possible explanations for the lower alpha diversity detected. First, redundancy of metabolisms in the bacterial community due to a pool of phylogenetically different community members capable to degrade the same substrates, which are not all active at the same time [2, 16, 91]. Second, community resilience, with other members in dormant stages that allow further functional adaptations when the environmental conditions change [16, 91]. Third, differences in the copy numbers of 16 S rRNA genes between taxa, inflating the abundance of a taxa but not portraying the actual functional scenario [15, 16, 91]. However, the entire and active community are not significantly different and follow similar temporal fluctuations. Therefore, studying only the entire community provides insights into the temporal fluctuations of the gut microbiome, but studying the active community indicated functionally important active taxa can go unnoticed because of their lower abundances at entire community level. Regarding the most abundant genera detected differing from the entire community, there is no information about the metabolism of *Colidextribacter*, while *Collinsella* and *Oribacterium* are polysaccharide degraders coinciding with the lemurs’ diet [92–94].

The functional predictions from the active community indicated an increase in fermentation and chemoheterotrophy during the rainy season possibly associated to the higher consumption of fruits and flowers [8, 9, 12, 14]. However, we did not detect an augmentation of cellulolytic metabolism correlating with leaf consumption during the dry season. Since we performed metabolic predictions from taxonomy, we consider this is caused by the limited and biased metabolic information for certain taxa.

### Dietary changes have an effect in the temporal fluctuations of the gut microbiome

The collection of behavioral data and the dietary assessment performed with the 18 S rRNA gene data allowed us to confirm temporal fluctuations of the gut microbiome correlate to dietary changes. We detected differentially abundant taxa for the rainy season, correlations of flower and fruit consumption to the temporal variations in β-diversity, and taxon-specific effects of flower and fruit consumption in bacterial composition. Flowers and fruits are high in non-structural polysaccharides like mono- and disaccharides, but flowers contain more protein whereas fruits have a higher lipid content [95–97]. The positively affected taxa by the consumption of these plant parts coincide with these observations, since they are reported fermenters of mono- and disaccharides, like *Succinivibrio, Oscillospiraceae,* and *Prevotellaceae*, while *Anaerovibrio*, metabolizes glycolipids [85, 98–100]. Furthermore, *Succinivibrio* and *Anaerovibrio* produce succinate from their fermentations which in turn is the energy source of *Phascolarctobacterium*, another positively affected taxon [98, 99, 101]. The correlation of flower consumption with a lower alpha diversity suggests that a diverse gut microbial community is not needed for the digestion of flowers, coinciding with their less complex biochemical composition [96, 97].

Against our expectations, consumption of leaves only correlated to β-diversity changes from the dry season but did not influence the overall bacterial composition. However, leaf consumption correlated to higher alpha diversities, also reported in the Hadza community and baboons [11, 14]. As leaves have complex structural polysaccharides like hemicellulose, cellulose, and lignin, this indicates that a more diverse bacterial community is needed for the processing of the structural polysaccharides from a leaf diet [97].

### Social interactions have an effect in the temporal fluctuations of the gut microbiome

Affiliative interactions correlated to the changes in β-diversity and influenced the overall gut microbiome composition. Lemurs use their toothcomb to groom themselves and others, by doing so, they can uptake microorganisms present on their furs and anogenital regions [102]. *Rikenellaceae, Alloprevotella, Kiritimatiellae*—WCHB1–41, and *Spirochaetaceae* were positively affected by affiliative interactions, indicating that they are transmitted via affiliative interactions. Social interactions correlated with β-diversity fluctuations in the dry season as well. During this period, social behaviors like mating, birth, and social thermoregulation to cope with the low temperatures occur, increasing microbe transmission [19, 103]. Nonetheless, there were no births during our study period suggesting social thermoregulation played a more important role [19].

### Correlations between precipitation and temporal fluctuations of the gut microbiome

Precipitation correlated with the fluctuations in β-diversity and a lower alpha diversity during the rainy season. Redfronted lemurs drink from waterholes and temporary ponds during the rainy season, whereas in the dry season, they drink from partially dry water holes in the river having higher microbial loads [20]. Higher precipitation resulting in water sources with lower microbial loads decreased alpha diversity and correlated to changes in β-diversity. Thus, taxa with higher abundances in the dry season could be ingested from drinking at the river waterholes like *Kiritimatiellae*—WCHB1–41, which was impacted negatively by higher precipitation and has been previously isolated from environmental water suggesting transmission from water sources [104].

### Gut of redfronted lemurs is inhabited by a great diversity of molecularly uncharacterized helminths and protozoa

The gut of all individuals harbored helminth and protozoan organisms over the entire year. These were classified only at the order level because they had high identity but low coverage to parasites of humans or livestock at higher taxonomical resolution. We detected a high prevalence of the *Chromadorea* and suspect most are from *Lemuricola vauceli* or *Callistoura* of *Oxyuridae*; however, genetical information from the V4 region of these organisms is absent in databases [18, 22]. This high prevalence has been previously detected morphologically but not in other metabarcoding studies [18, 22, 23]. Furthermore, our phylogenetic analysis detected other families like *Trichuridae* and *Strongyloididae* confirming a great diversity of nematodes inhabiting these lemurs [22].

The sequences detected from *Trichostomatia* possibly belong to *Balantidium*, following previous microscopical reports of this lemur species [22, 23]. Moreover, the identified *Trichomonadida*, possibly a novel organism, was not detected before in microscopical studies, only in amplicon-based reports [22, 23]. The differences in the taxa detected between this study and a previous metabarcoding report might be due to the amplification of different regions of the 18 S rRNA gene, we used the V4 while in other studies the V3-V4 and V3-V5 were investigated [23, 31].

### The gut mycobiome of redfronted lemurs is comprised by novel fungi

We detected in low relative abundances fungal genera described as human gut symbionts, suggesting the gut mycobiome of redfronted lemurs has low abundances and diversity, as reported in humans [24]. The majority of the ASVs were unclassifiable, even after performing a phylogenetic analysis of the most abundant sequences against representative fungi, confirming the deficiency in genomic information from fungal organisms found in Madagascar and the gut of wild-living animals [24, 82]. The observed variation of the unclassified taxa between months could portray changes in the gut mycobiome. Nonetheless, it should be considered that some of the detected taxa might derive from diet, as redfronted lemurs fed on fungi or fungal plant pathogens [25, 82].

## Conclusion

Fruit and flower consumption, affiliative interactions and water sources are important drivers of the temporal fluctuations of the gut bacterial communities from redfronted lemurs. Thus, displaying how this bacterial community adapts to the host diet and behavior following temporal changes. Eukaryotic gut communities also fluctuate monthly and are very diverse. Our results affirm intricate host-microbiome interactions in the gut of redfronted lemurs are affected by the host diet, precipitation, and social behavior. To our knowledge, this is the first 1-year study combining thorough sampling and individual behavioral data collection allowing the detection of direct links between temporal fluctuations of bacterial taxa and consumption of specific food items and social behavior. Longitudinal studies as the one performed here capture better the effects of seasonality on the fluctuations of the gut microbiome, diet, and social behaviors than cross-sectional approaches.

## Supplementary information

Supplementary Tables S1–S14

Supplementary Figures S1–S16
