# Supplementary material for: Dietary shifts and social interactions drive temporal fluctuations of the gut microbiome from wild redfronted lemurs
Source: ISME Commun. 2022 Jan 19;2:3. doi: 10.1038/s43705-021-00086-0 (PMC9723586; doi:10.1038/s43705-021-00086-0)
Supplement: Supplementary file 2 — Supplementary Figures S1–S16 [file 43705_2021_86_MOESM2_ESM.pdf]

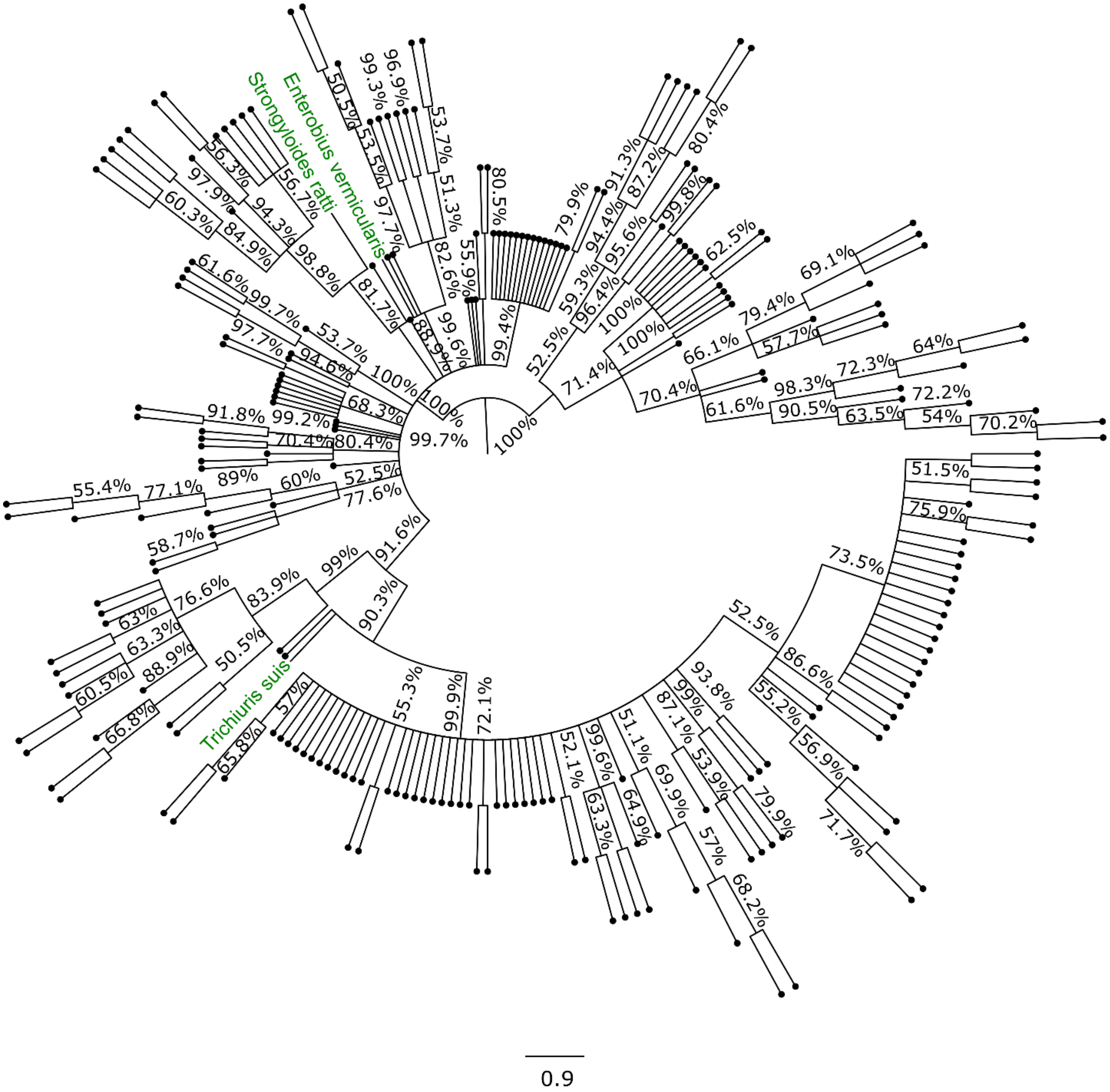

Supplemental Figure S1. Maximum likelihood phylogenetic tree of the ASVs classified as *Chromadorea* including representative parasitic nematodes of humans and animals. Sequences were aligned with MUSCLE with UPGMA and default settings, and the phylogenetic tree was generated in MEGA X with the Tamura-Nei model and 1 000 bootstrap. 18S rRNA gene sequences from representative parasitic nematodes of humans and animals were retrieved from the NCBI database.

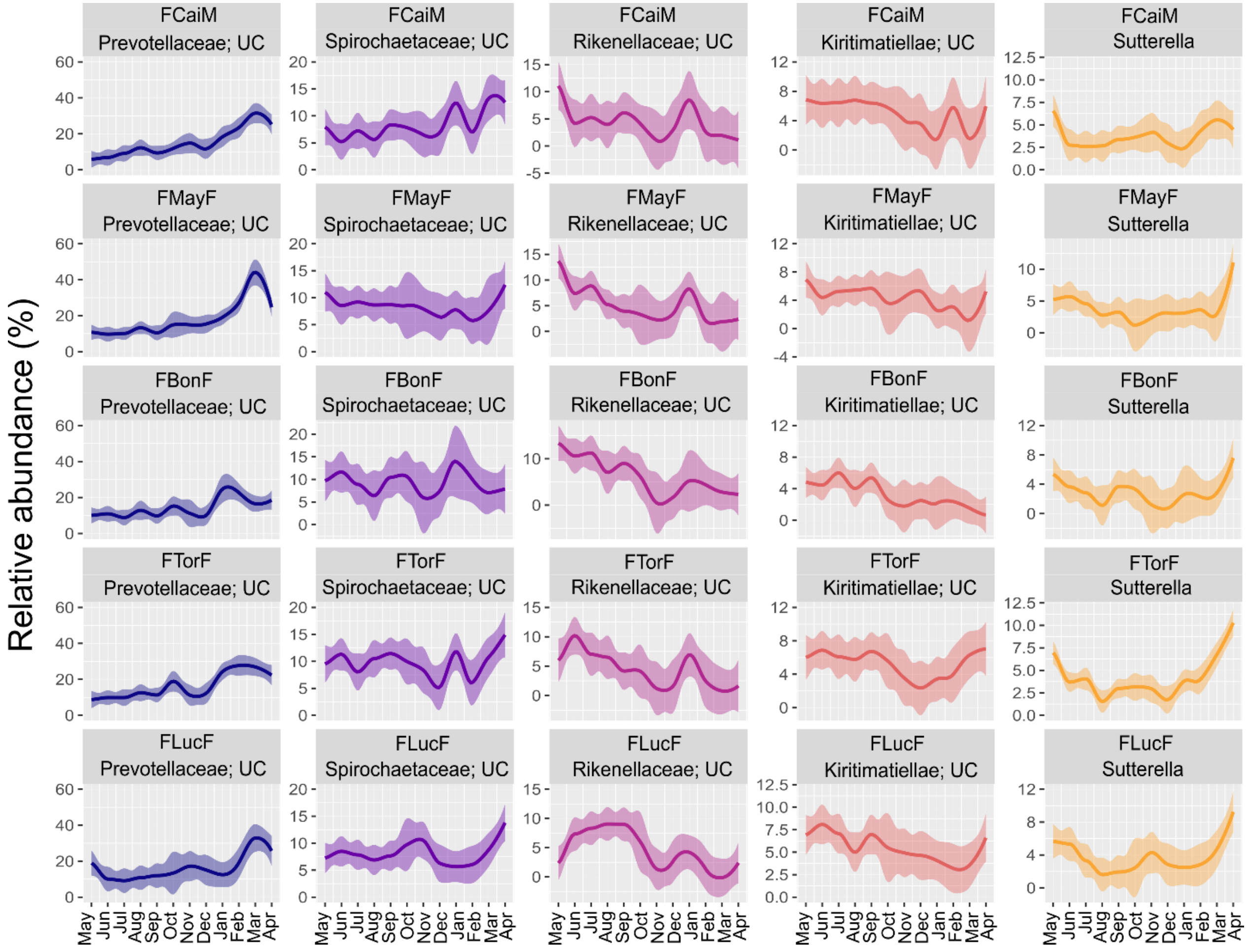

Supplemental Figure S2. Monthly fluctuations of the five most abundant bacterial genera in each individual. Linecharts depict relative abundances of bacterial genera from normalized counts.

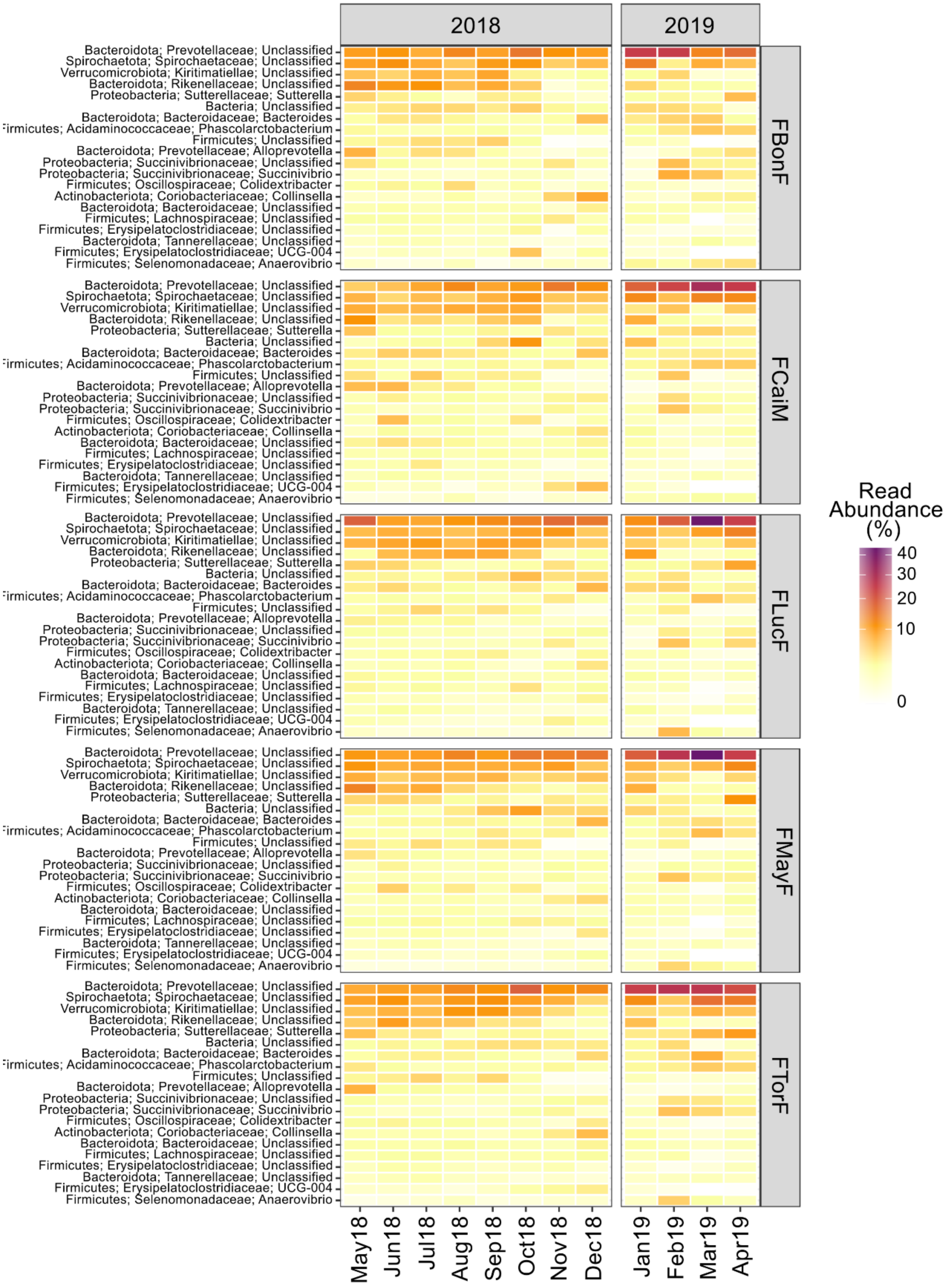

Supplemental Figure S3. Heatmap of the 20 most abundant bacterial genera averaged per month for each individual during the study period. Relative abundances were estimated from normalized counts.

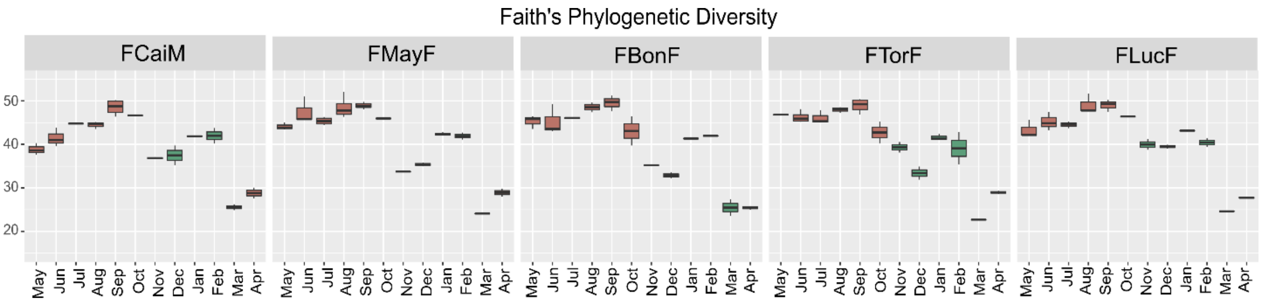

Supplemental Figure S4. Monthly individual fluctuations in alpha diversity measured as Faith's Phylogenetic diversity index.

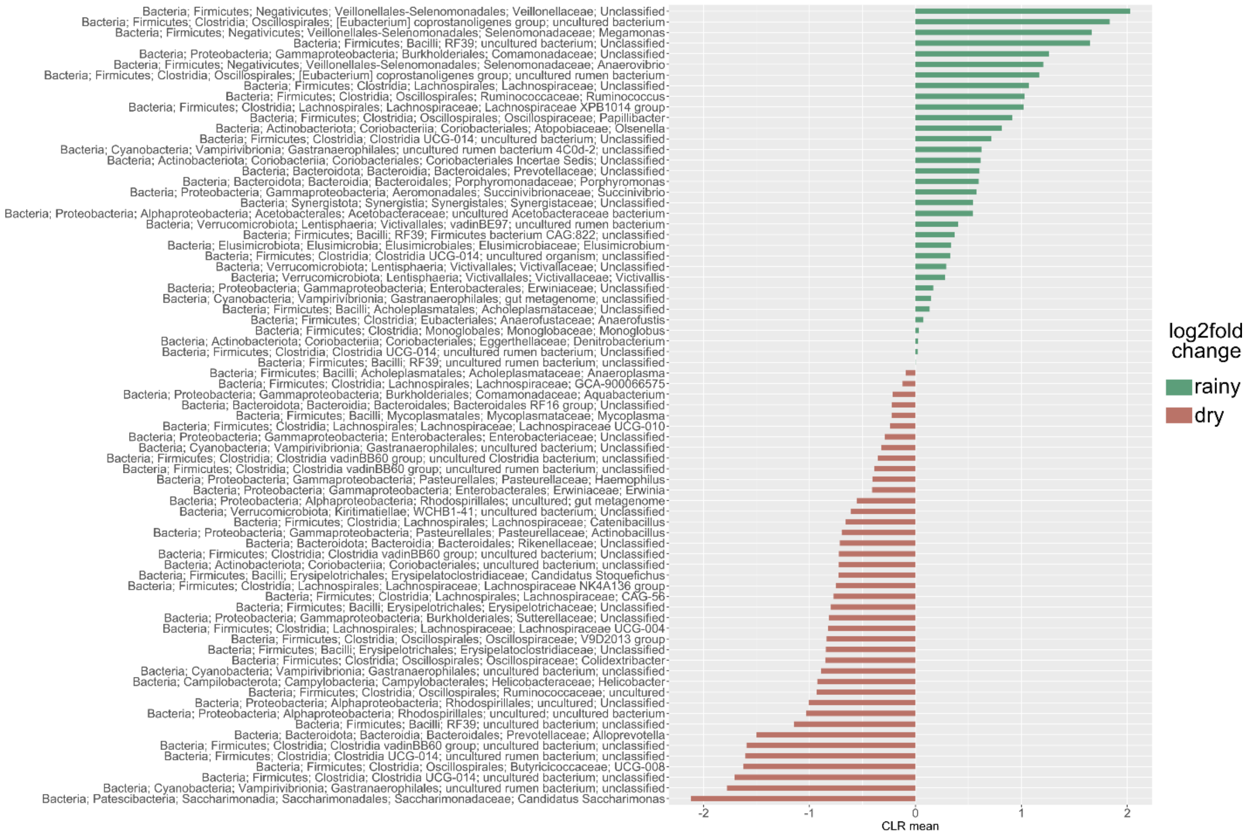

Supplemental Figure S5. Log2fold changes of mean abundances of bacterial genera between dry and rainy season as detected with ANCOM 2.1.

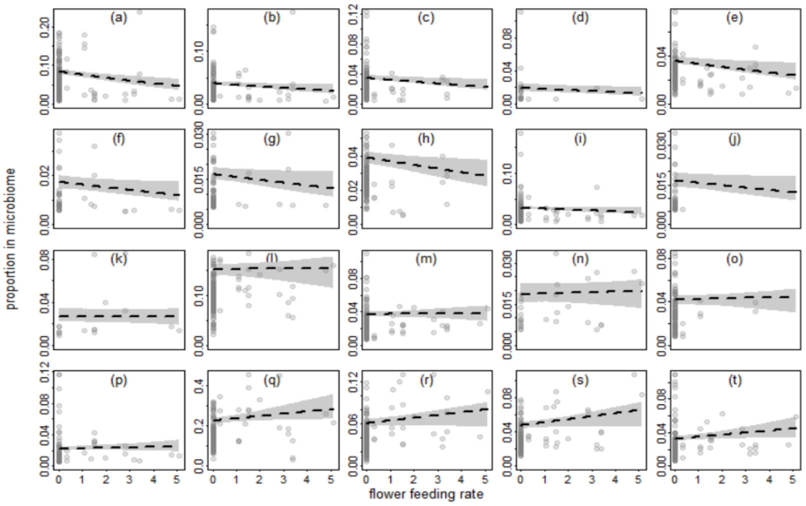

Subplot	Taxon
a	Rikenellaceae:Unclassified
b	Prevotellaceae:Alloprevotella
c	Oscillospiraceae:Colidextribacter
d	Veillonellaceae:Unclassified
e	Bacteroidaceae:Unclassified
f	Eggerthellaceae:uncultured
g	Oscillospiraceae:UCG -005
h	Lachnospiraceae:uncultured
i	Coriobacteriaceae:Collinsella
j	Prevotellaceae:UCG -004
k	Coriobacteriaceae:Coriobacterium
l	Spirochaetaceae:Unclassified
m	Succinivibrionaceae:uncultured
n	Akkermansiaceae:Akkermansia
o	uncultured:Unclassified
p	Selenomonadaceae:Anaerovibrio
q	Prevotellaceae:Unclassified
r	Sutterellaceae:Sutterella
s	Acidaminococcaceae:Phascolarctobacterium
t	Succinivibrionaceae:Succinivibrio

Supplemental Figure S6. Effects of flower consumption rates on the 20 bacterial taxa for which the taxon-specific effect differed most from the average effect across all taxa. LMMs were calculated including the random intercept of individual, taxon, sample, and taxon nested within individual (taxon-individual) and the random slope and fixed effect of monthly rates of flower feeding. A significant effect of flower feeding on community composition was determined by comparing the log likelihoods of the full model to one lacking the random slopes of all test predictors within taxon, and another one without flower feeding rates within taxon. Additionally, 1 000 permutations were performed by shuffling the labels of taxa within sample to detect a specific effect of flower feeding in a bacterial taxon. Significance was determined as the proportion of permutations where the test statistic was at least as large as the original data.

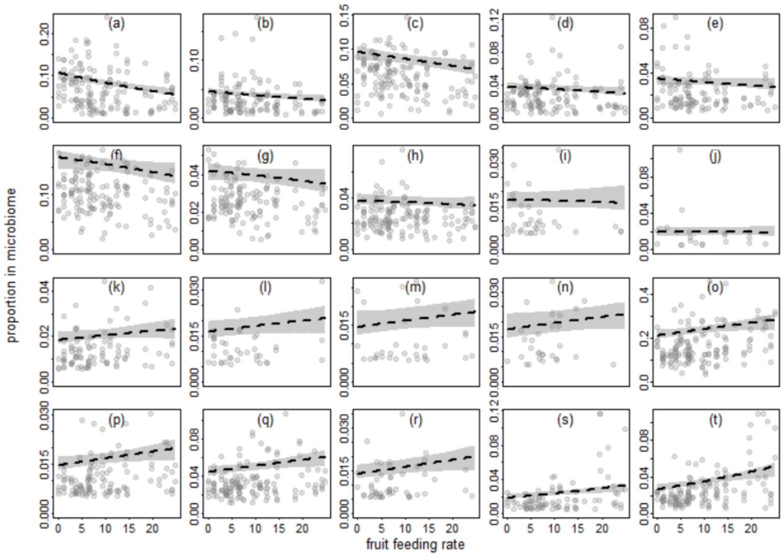

Subplot	Taxon
a	Rikenellaceae:Unclassified
b	Prevotellaceae:Alloprevotella
c	Oscillospiraceae:Colidextribacter
d	Veillonellaceae:Unclassified
e	Bacteroidaceae:Unclassified
f	Eggerthellaceae:uncultured
g	Oscillospiraceae:UCG -005
h	Lachnospiraceae:uncultured
i	Coriobacteriaceae:Collinsella
j	Prevotellaceae:UCG -004
k	Coriobacteriaceae:Coriobacterium
l	Spirochaetaceae:Unclassified
m	Succinivibrionaceae:uncultured
n	Akkermansiaceae:Akkermansia
o	uncultured:Unclassified
p	Selenomonadaceae:Anaerovibrio
q	Prevotellaceae:Unclassified
r	Sutterellaceae:Sutterella
s	Acidaminococcaceae:Phascolarctobacterium
t	Succinivibrionaceae:Succinivibrio

Supplemental Figure S7. Effects of fruit consumption rates on the 20 bacterial taxa for which the taxon-specific effect differed most from the average effect across all taxa. LMMs were calculated including the random intercept of individual, taxon, sample, and taxon nested within individual (taxon-individual) and the random slope and fixed effect of monthly rates of fruit feeding. A significant effect of fruit feeding on community composition was determined by comparing the log likelihoods of the full model to one lacking the random slopes of all test predictors within taxon, and another one without fruit feeding rates within taxon. Additionally, 1 000 permutations were performed by shuffling the labels of taxa within sample to detect a specific effect of fruit feeding in a bacterial taxon. Significance was determined as the proportion of permutations where the test statistic was at least as large as the original data.

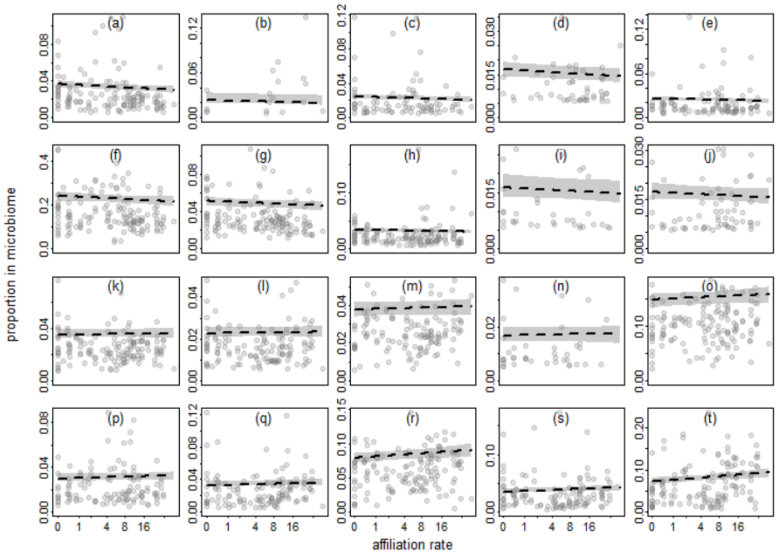

Subplot	Taxon
a	Rikenellaceae:Unclassified
b	Prevotellaceae:Alloprevotella
c	Oscillospiraceae:Colidextribacter
d	Veillonellaceae:Unclassified
e	Bacteroidaceae:Unclassified
f	Eggerthellaceae:uncultured
g	Oscillospiraceae:UCG -005
h	Lachnospiraceae:uncultured
i	Coriobacteriaceae:Collinsella
j	Prevotellaceae:UCG -004
k	Coriobacteriaceae:Coriobacterium
l	Spirochaetaceae:Unclassified
m	Succinivibrionaceae:uncultured
n	Akkermansiaceae:Akkermansia
o	uncultured:Unclassified
p	Selenomonadaceae:Anaerovibrio
q	Prevotellaceae:Unclassified
r	Sutterellaceae:Sutterella
s	Acidaminococcaceae:Phascolarctobacterium
t	Succinivibrionaceae:Succinivibrio

Supplemental Figure S8. Effects of affiliation rates on the 20 bacterial taxa for which the taxon-specific effect differed most from the average effect across all taxa. LMMs were calculated including the random intercept of individual, taxon, sample, and taxon nested within individual (taxon-individual) and the random slope and fixed effect of monthly affiliation rates. A significant effect of affiliation rates on community composition was determined by comparing the log likelihoods of the full model to one lacking the random slopes of all test predictors within, and another one without affiliation rates within taxon. Additionally, 1 000 permutations were performed by shuffling the labels of taxa within sample to detect a specific effect of affiliation rates in a bacterial taxon. Significance was determined as the proportion of permutations where the test statistic was at least as large as the original data.

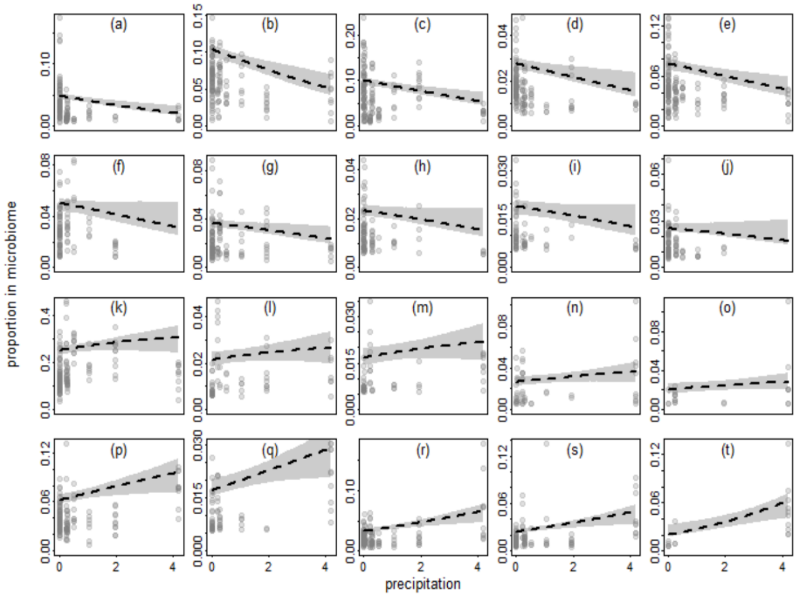

Subplot	Taxon
a	Prevotellaceae:Alloprevotella
b	Kiritimatiellae:WCHB1-41
c	Rikenellaceae:Unclassified
d	Tannerellaceae:Unclassified
e	Sutterellaceae:Sutterella
f	Clostridia:UCG-14
g	Kiritimatiellae:WCHB1-41
h	Erysipelotrichaceae:Unclassified
i	Lachnospiraceae:Syntrophococcus
j	Clostridia:UCG-14
k	Prevotellaceae:Unclassified
l	Lachnospiraceae:Unclassified
m	Oscillospiraceae:Unclassified
n	Selenomonadaceae:Megamonas
o	Veillonellaceae:Unclassified
p	Bacteroidaceae:Bacteroides
q	Oscillospiraceae:UCG-5
r	Coriobacteriaceae:Collinsella
s	Erysipelatoclostridiaceae:UCG-4
t	Atopobiaceae:Olsenella

Supplemental Figure S9. Effects of mean precipitation on the 20 bacterial taxa for which the taxon-specific effect differed most from the average effect across all taxa. LMMs were calculated including the random intercept of individual, taxon, sample, and taxon nested within individual (taxon-individual) and the random slope and fixed effects of the test predictors: feeding rates of flowers, fruits and leaves, and affiliation rates. Precipitation was included as a control predictor, thus taxon-specific were not tested for significance.

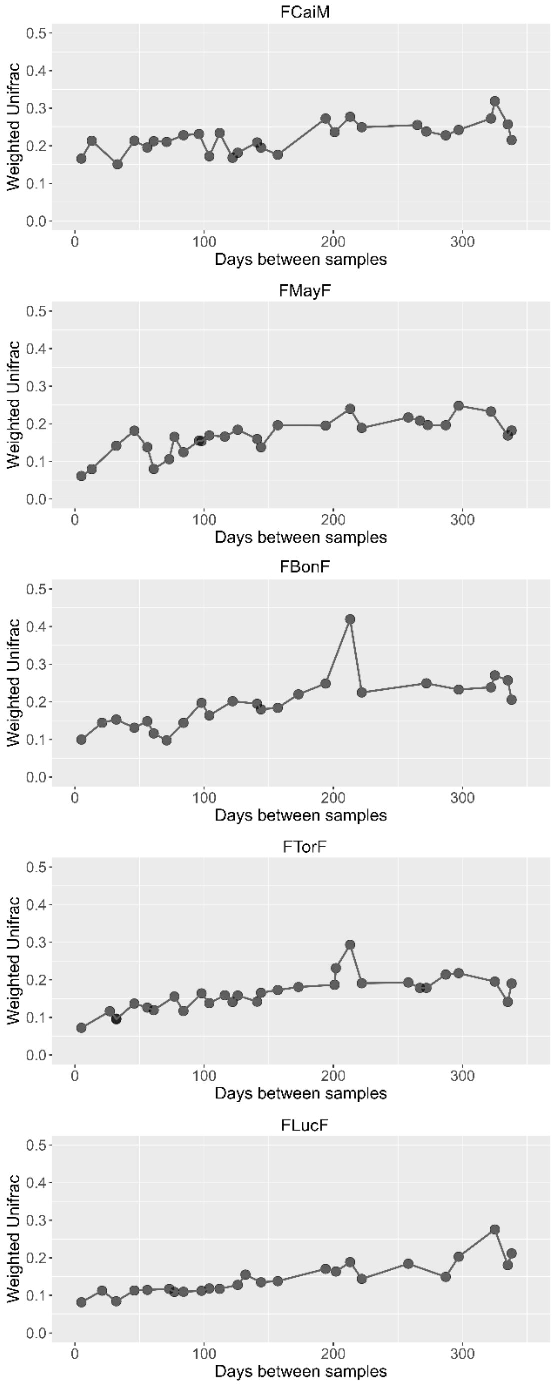

Supplemental Figure S10. Time decay plots of β-dissimilarities against the time span between sample collection for each individual. Wunifrac distance matrices for each individual were calculated and compared to matrices accounting for the days between sample collection. Mantel tests were used to determine correlations between sample dissimilarities and sampling interval.

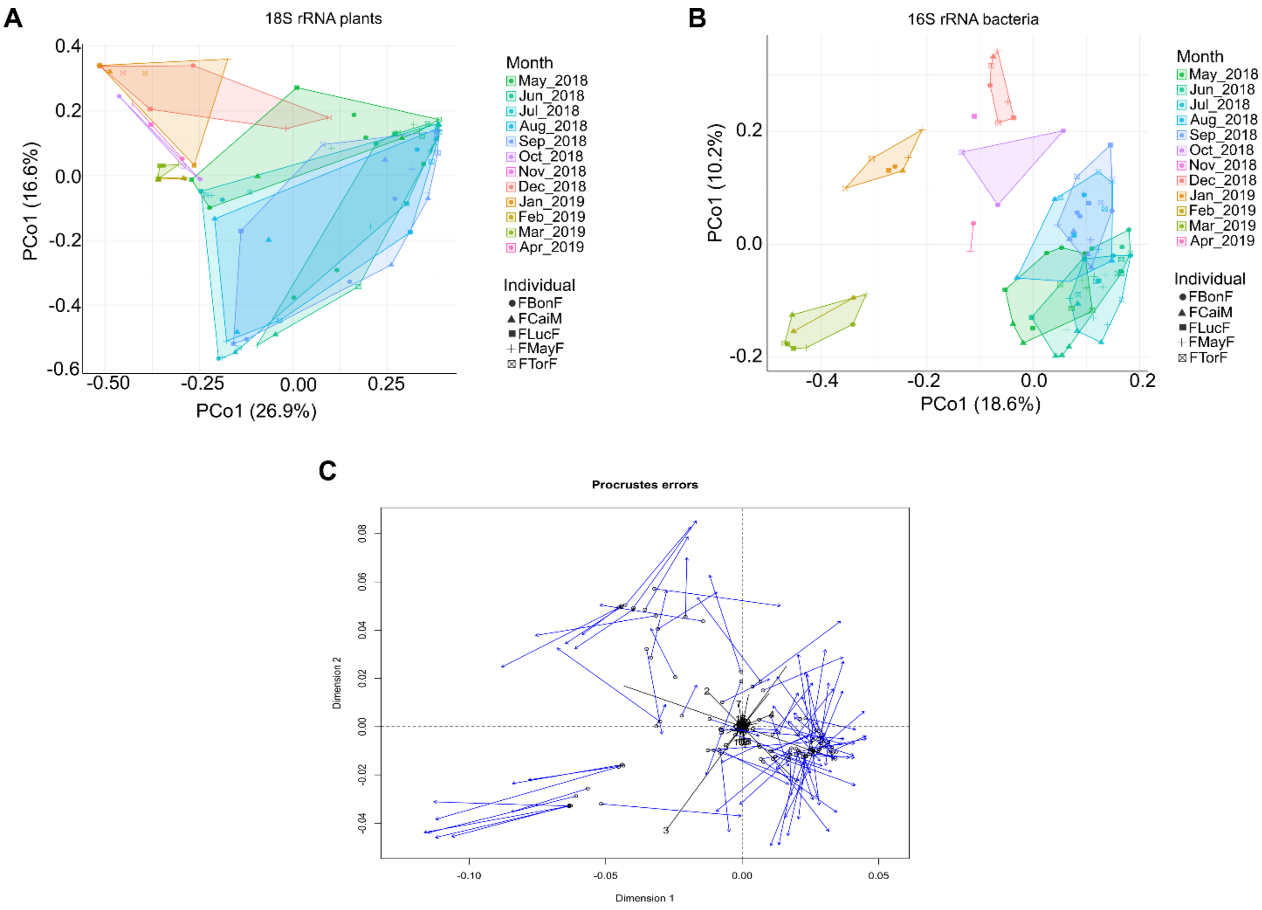

Supplemental Figure S11. Comparison between the monthly variations in gut microbiome composition and dietary items. **A.** PCoA from Bray Curtis dissimilarity distance of the dietary items detected from the 18S rRNA amplicon sequencing. **B.** PCoA from Bray Curtis dissimilarity distances of the entire bacterial community and its temporal variations. **C.** Procrustes comparison of the ordination analysis for the bacteria and dietary items identified.

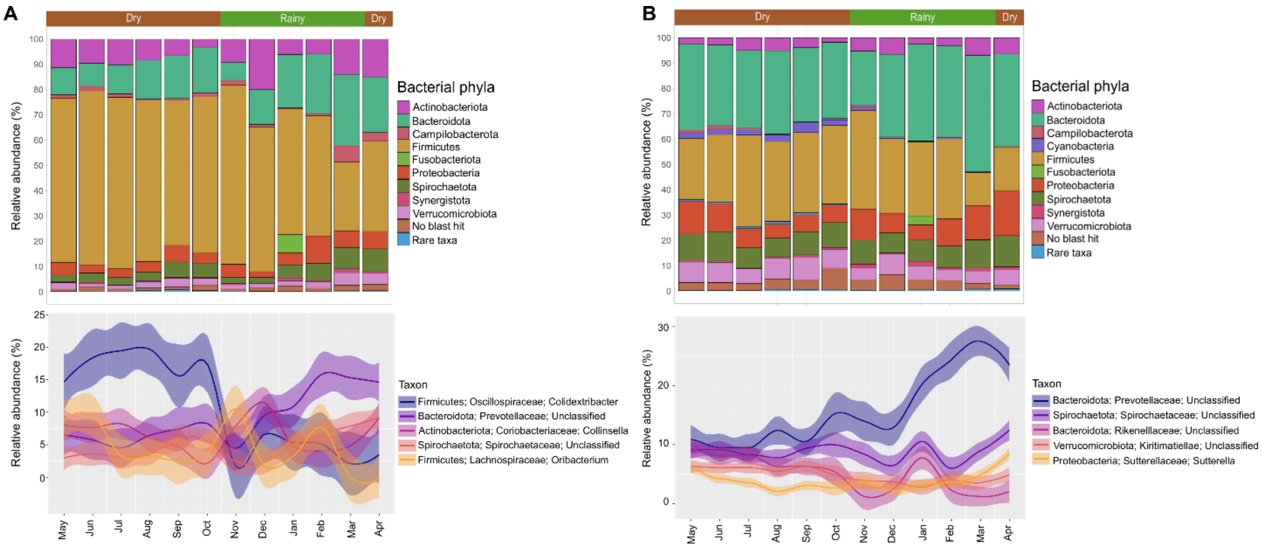

Supplemental Figure S12. Comparison of the bacterial composition to phyla level and the five most abundant genera for the **A.** active community and **B**. entire community and their monthly fluctuations from May 2018 to April 2019. Barcharts to phylum level and linecharts to genus level display relative abundances from normalized counts.

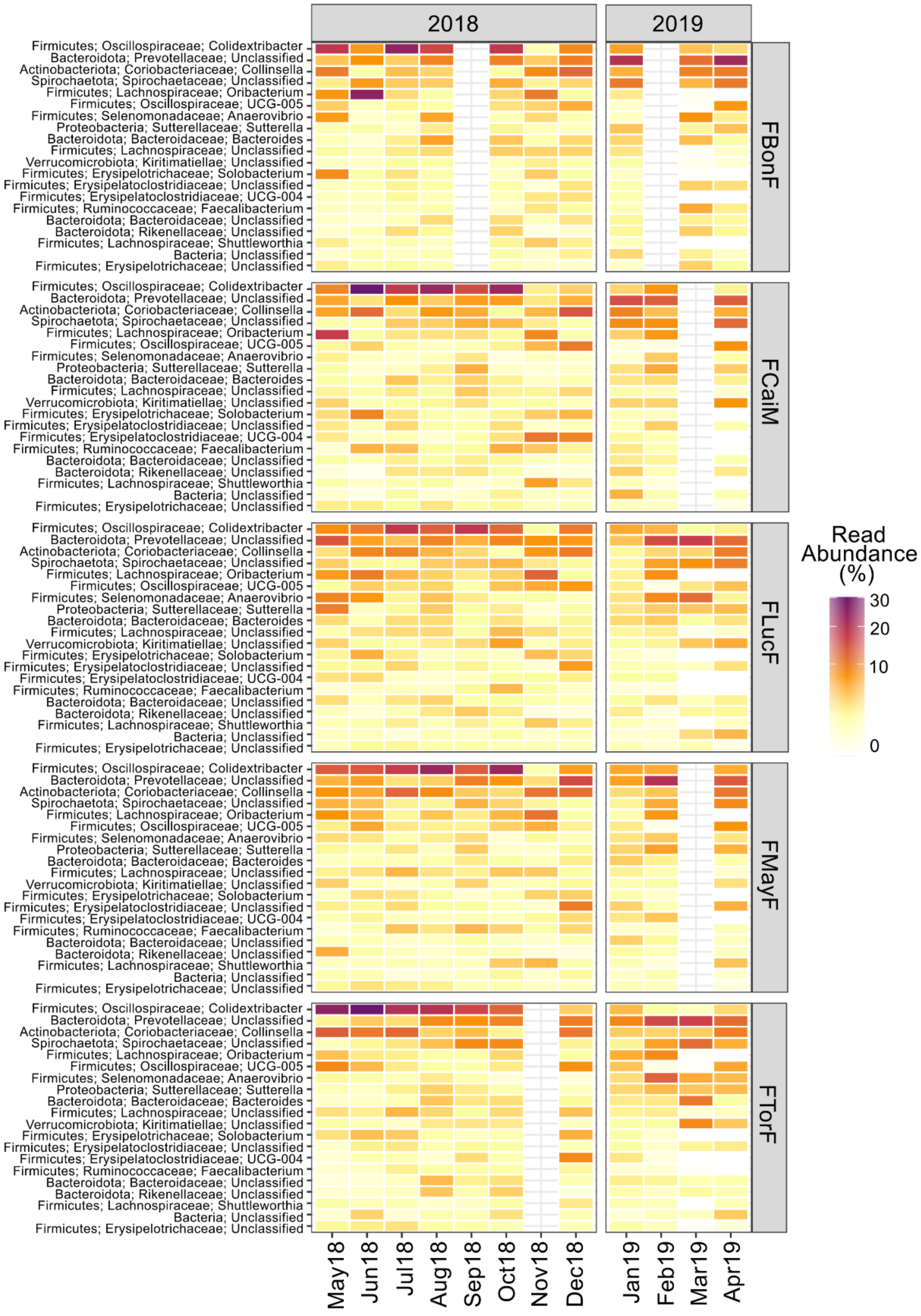

Supplemental Figure S13. Heatmap of the 20 most abundant bacterial genera in the potential active community and their monthly fluctuations in their relative abundances in each individual. Relative abundances were estimated from normalized counts.

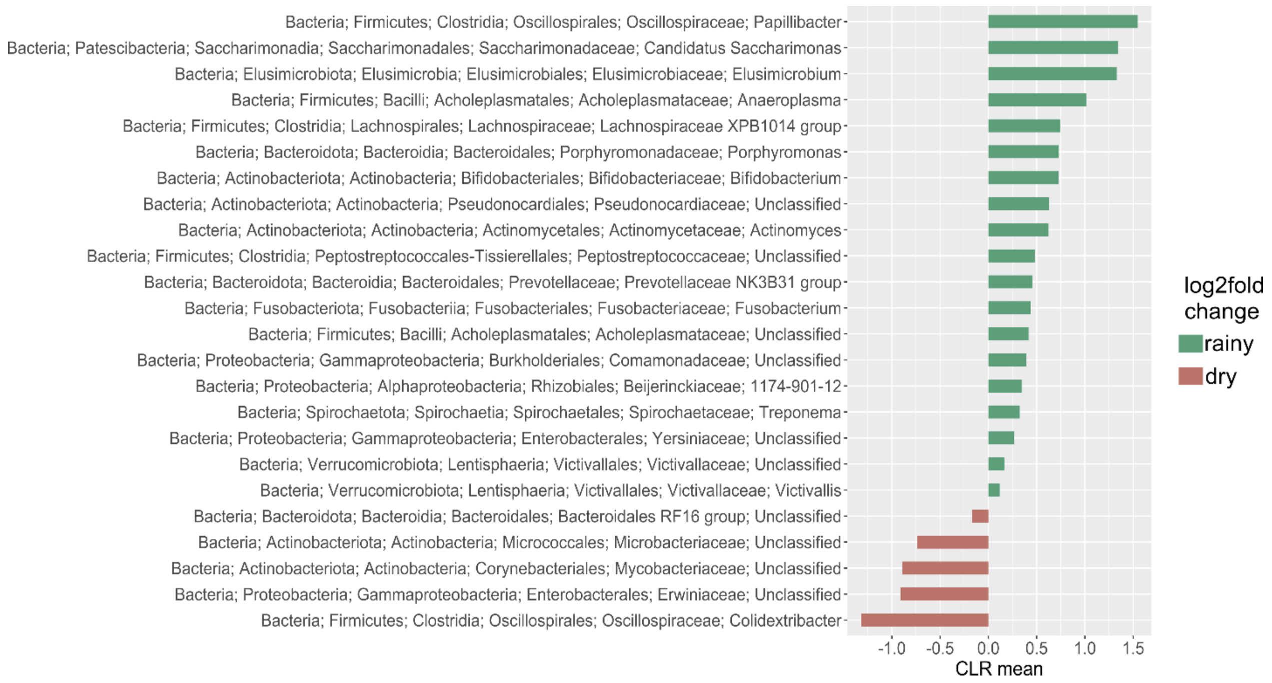

Supplemental Figure S14. Log2fold changes in the mean abundances of bacterial genera between dry and rainy season in the potential active community as detected with ANCOM 2.1.

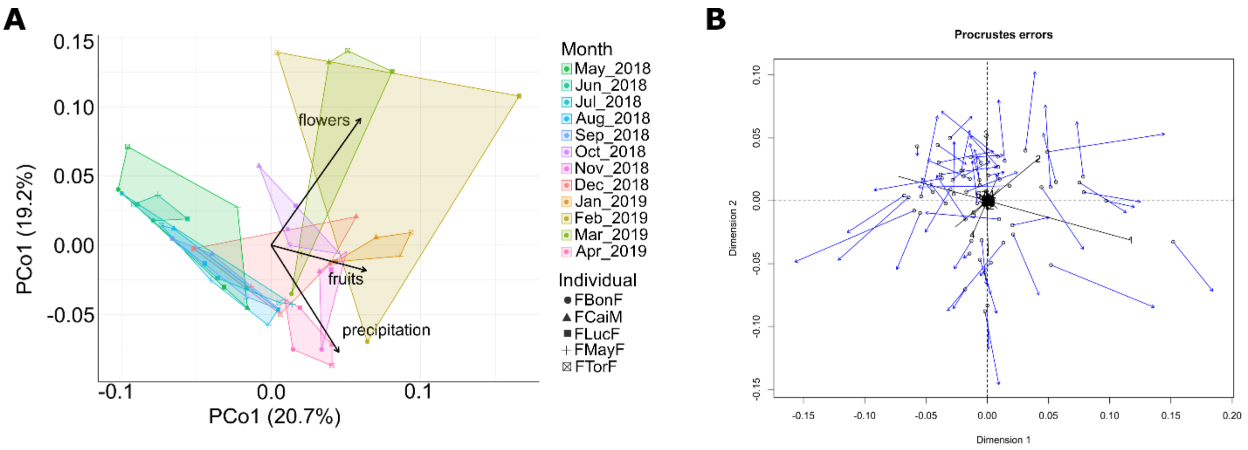

Supplemental Figure S15. Comparison between the entire and the active bacterial community. **A.** PCoA from WUnifrac of the reduced entire community and the dietary and environmental fit analysis depicting significant correlations between temporal fluctuations and the environmental, diet and social factors investigated. **B**. Results from the Procrustes comparison of the PCoAs from Wunifrac for the bacteria potential active and entire community.

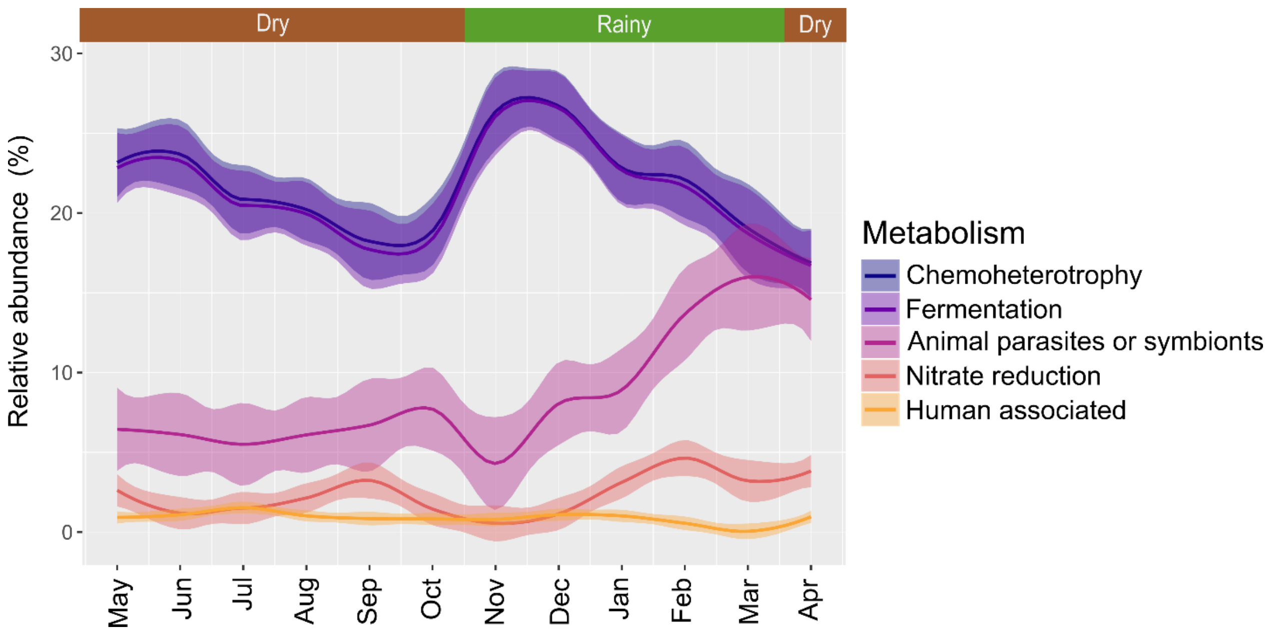

Supplemental Figure S16. Functional predictions for the active bacterial community using Faprotax.
